# Thioredoxin Reductase 2 (*TXNRD2*) Mutation Associated With Familial Glucocorticoid Deficiency (FGD)

**DOI:** 10.1210/jc.2013-3844

**Published:** 2014-03-06

**Authors:** Rathi Prasad, Li F. Chan, Claire R. Hughes, Juan P. Kaski, Julia C. Kowalczyk, Martin O. Savage, Catherine J. Peters, Nisha Nathwani, Adrian J. L. Clark, Helen L. Storr, Louise A. Metherell

**Affiliations:** Centre for Endocrinology (R.P., L.F.C., C.R.H., J.C.K., M.O.S., A.J.L.C., H.L.S., L.A.M.), Queen Mary University of London, William Harvey Research Institute, Barts and the London School of Medicine and Dentistry, London EC1M 6BQ, United Kingdom; Inherited Cardiovascular Diseases Unit (J.P.K.), Department of Cardiology, Great Ormond St Hospital for Children, and Department of Paediatric Endocrinology (C.J.P.), Great Ormond St Hospital for Children, London WC1N 3JH, United Kingdom; and Department of Paediatric Endocrinology (N.N.), Luton and Dunstable University Hospital, Luton LU4 0DZ, United Kingdom

## Abstract

**Context:**

Classic ACTH resistance, due to disruption of ACTH signaling, accounts for the majority of cases of familial glucocorticoid deficiency (FGD). Recently FGD cases caused by mutations in the mitochondrial antioxidant, nicotinamide nucleotide transhydrogenase, have highlighted the importance of redox regulation in steroidogenesis.

**Objective:**

We hypothesized that other components of mitochondrial antioxidant systems would be good candidates in the etiology of FGD.

**Design:**

Whole-exome sequencing was performed on three related patients, and segregation of putative causal variants confirmed by Sanger sequencing of all family members. A *TXNRD2-*knockdown H295R cell line was created to investigate redox homeostasis.

**Setting:**

The study was conducted on patients from three pediatric centers in the United Kingdom.

**Patients:**

Seven individuals from a consanguineous Kashmiri kindred, six of whom presented with FGD between 0.1 and 10.8 years, participated in the study.

**Interventions:**

There were no interventions.

**Main Outcome Measure:**

Identification and functional interrogation of a novel homozygous mutation segregating with the disease trait were measured.

**Results:**

A stop gain mutation, p.Y447X in *TXNRD2*, encoding the mitochondrial selenoprotein thioredoxin reductase 2 (TXNRD2) was identified and segregated with disease in this extended kindred. RT-PCR and Western blotting revealed complete absence of TXNRD2 in patients homozygous for the mutation. TXNRD2 deficiency leads to impaired redox homeostasis in a human adrenocortical cell line.

**Conclusion:**

In contrast to the *Txnrd2*-knockout mouse model, in which embryonic lethality as a consequence of hematopoietic and cardiac defects is described, absence of TXNRD2 in humans leads to glucocorticoid deficiency. This is the first report of a homozygous mutation in any component of the thioredoxin antioxidant system leading to inherited disease in humans.

Familial glucocorticoid deficiency (FGD; Mendelian Inheritance in Man 202200) is characterized by ACTH resistance and isolated glucocorticoid deficiency, with typical biochemical findings of low serum cortisol levels and high plasma ACTH (1). Patients commonly present with hyperpigmentation, a consequence of overstimulation of melanocortin 1 receptor by proopiomelanocortin products and hypoglycemia, which, if untreated, can lead to neurological sequelae. Clinical presentation includes failure to thrive in the neonatal or early childhood periods and an increased susceptibility to infections (1). This disorder can lead to significant morbidity and is potentially fatal if untreated. Classical ACTH resistance is primarily caused by mutations in the ACTH receptor [melanocortin 2 receptor (MC2R)] and the MC2R accessory protein (MRAP), required for trafficking MC2R to the cell surface and subsequent signaling (2, 3). Mutations in *MC2R* and *MRAP* thus disrupt ACTH signaling in the zona fasciculata, resulting in isolated glucocorticoid deficiency.

Our group recently described two novel pathogenic mechanisms for FGD (4, 5). A mutation in the DNA helicase, minichromosome maintenance 4 (*MCM4*) is responsible for FGD in the Irish traveler population, incorporating a phenotype of short stature, natural killer cell deficiency, and chromosomal fragility (4). Additionally, mutations in *NNT*, encoding the mitochondrial antioxidant nicotinamide nucleotide transhydrogenase, account for 10% of cases in our FGD cohort (5). Nicotinamide nucleotide transhydrogenase (NNT), located in the inner mitochondrial membrane, provides the high concentrations of reduced nicotinamide adenine dinucleotide phosphate (NADPH) required by the thioredoxin and glutathione systems to detoxify mitochondrial H_2_O_2_.

In this study, we describe the first homozygous mutation in the mitochondrial selenoprotein, thioredoxin reductase 2 (*TXNRD2*) associated with FGD in an extended consanguineous Kashmiri kindred (Figure 1A).

**Figure 1.**
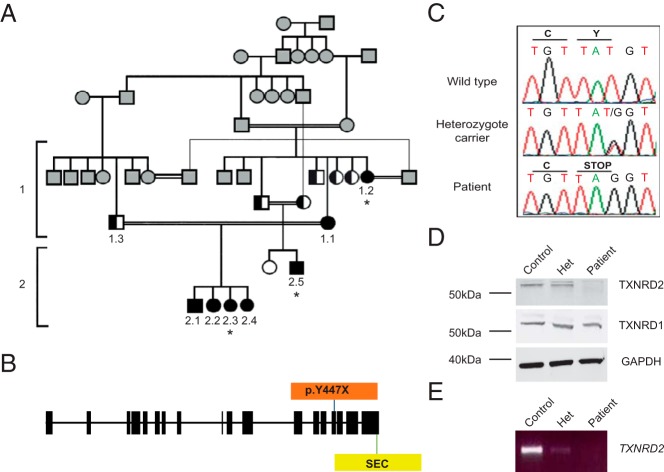
Pedigree of the affected kindred and identification of p.Y447X *TXNRD2* mutation leading to the loss of TXNRD2 protein. A, Pedigree of affected patients. Black-filled symbols indicate individuals homozygous and half-filled indicate individuals heterozygous for the mutation. White-filled symbols indicate wild-type individuals. Gray-filled symbols indicate individuals not tested. The asterisks denote the three affected individuals who were subjected to whole-exome sequencing. B, Gene structure of *TXNRD2*; p.Y447X mutation prior to the selenocysteine active site (SEC). C, Partial sequence chromatograms of genomic DNA from a wild-type, heterozygote carrier and a patient, showing the base change from T to G in exon 15, resulting in a premature stop codon in the affected individual. D, Lysates from a homozygous patient, heterozygote carrier, and control human lymphocytes were immunoblotted with an anti-TXNRD2 antibody. Although the control and the heterozygote carriers expressed the 56-kDa protein, this is absent in the homozygote patient, with no evidence of a truncated protein. All individuals express cytoplasmic TXNRD1 normally. E, RT-PCR of cDNA from the patient, heterozygote carrier, and control suggested nonsense mediated decay of mRNA.

## Materials and Methods

### Study approval

This study was approved by the Outer North East London Research Ethics Committee, reference number 09/H0701/12.

### Candidate gene sequencing

Genomic DNA was extracted from peripheral blood leukocytes of affected individuals and their family members after obtaining informed consent from them and/or their parents. Sequencing of coding exon/intron boundaries of *MC2R*, *MRAP*, *STAR*, and *NNT* genes had previously been undertaken by conventional Sanger sequencing and no mutations were found. The coding exons of *MCM4* and *TXNRD2* were sequenced in 50 patients with a clinical diagnosis of FGD by a combination of whole-exome and Sanger sequencing. Whole-exome sequencing data were analyzed in each individual and for any exonic region with a coverage of less than 20 reads. PCR amplification and Sanger sequencing was carried out. No mutations were found.

### Whole-exome sequencing

Whole-exome sequencing using the Illumina HiSeq 2000 sequencer was conducted on three affected individuals (samples processed by Otogenetics Corp). The whole-exome sequencing samples were prepared as an Illumina sequencing library, and in the second step, the sequencing libraries were enriched using the Agilent V4 enrichment kit. The captured libraries were sequenced and downstream analysis conducted via DNAnexus (see *Acknowledgments*). Single-nucleotide polymorphisms (SNPs), with a threshold coverage of at least 10 reads on the respective nucleotide, were assessed.

The number of variants was reduced by the following strategy: 1) identifying variants that were common to all three individuals; 2) excluding variants that were heterozygous; 3) removing variants, annotated in SNP databases (Ensembl SNP database, release 54), with a minor allele frequency of greater than 0.01; and 4) evaluating nonsynonymous coding variants, splice variants, and indels only. Finally, four candidate variants in three genes (*OR2T35*, *MUC4*, and *TXNRD2*) were investigated for the segregation of disease within the kindred by Sanger sequencing.

### PCR and sequencing

Each exon of the genes of interest including intronic boundaries was amplified by PCR using specific primers (primer sequences are listed in Supplemental Table 1). The reaction mixture contained 100 ng DNA template, 1× PCR buffer, 200 μM each deoxynucleotide triphosphate, 200 mM each primer, and 1 U *Taq* DNA polymerase (Sigma-Aldrich). Cycling conditions were as follows: 95°C for 5 minutes (one cycle); 95°C for 30 seconds, 55°C for 30 seconds, and 72°C for 30 seconds (30 cycles); and 72°C for 5 minutes. PCR products were visualized on 1% agarose gel and sequenced using the ABI Prism Big Dye sequencing kit and an ABI 3700 automated DNA sequencer (Applied Biosystems), in accordance with the manufacturer's instructions.

### RNA extraction and cDNA sequencing

Total RNA was isolated and purified using the PAXgene blood RNA (QIAGEN) system according to the manufacturer's instructions. The RNA was reverse transcribed and cDNA subsequently used as a template for PCR amplification and sequencing of *TXNRD2* exons 14–16 (primer sequences listed in Supplemental Table 1).

### Leukocyte separation for immunoblotting

Fresh whole blood was collected in EDTA-containing tubes. Mononuclear cells were extracted using a gradient density centrifugation method with Histopaque-1077 according to the manufacturer's protocol (Sigma-Aldrich). Cells were lysed with radioimmunoprecipitation assay buffer [50 mM Tris-HCL (pH 8.0), with 150 mM sodium chloride, 1% IGEPAL CA-630 (Nonidet P-40), 0.5% sodium deoxycholate, and 0. 1% sodium dodecyl sulfate], supplemented with complete, Mini, EDTA-free protease inhibitor cocktail tablets (Roche), placed on ice for 30 minutes, and centrifuged at 15 000 × *g* for 12 minutes at 4°C. The supernatant was subsequently added to an equal volume of Laemmli loading buffer. Protein expression of TXNRD2 in patient lysates was determined by immunoblotting with a rabbit polyclonal anti-TXNRD2 antibody (Sigma-Aldrich).

### In vitro adrenocortical model

#### Cell culture

H295R adrenocortical tumor cells were cultured in GIBCO DMEM/F12-Ham (1:1) + GlutaMAX-I, supplemented with 5% NuSerum, penicillin/streptomycin, and insulin-transferrin-selenium. HEK293T cells were maintained in DMEM/F12 (1:1) supplemented with 2% NuSerum and penicillin/streptomycin. All cells were incubated in a humidified incubator at 37°C and 5% CO_2_.

### Short hairpin RNA (shRNA) lentiviral transduction

Stable knockdown of *TXNRD2* was established in H295R human adrenocortical cells by lentiviral shRNA transduction. Lentiviral plasmids (V3LHS_354173) were obtained from OpenBiosystems in a p.GIPZ backbone and contained shRNA specific for human TXNRD2 (NM 10587) under the control of the cytomegalovirus promoter, plus the puromycin resistance and green fluorescence protein (GFP) genes. HEK293T cells (packaging cells) were transiently transfected with the shRNA plasmids together with packaging vectors, PMDG.2 plasmid and the cytomegalovirus plasmid 8.74 plasmid, using Lipofectamine 2000 (Invitrogen Life Technologies) as per the manufacturer's guidelines. Two days after transfection, virus-containing media were collected, filtered using a 0.22-μm filter and used to transduce H295R cells. Five days after infection, GFP-positive cells were selected in 5 μg/mL puromycin. Transduction efficiency was determined by fluorescence microscopy. A scrambled (control) cell line was generated using a lentiviral plasmid vector containing a shRNA insert that does not target human and mouse genes (Open Biosystems).

### Immunoblotting

An anti-TXNRD2 antibody was used to assess patient TXNRD2 expression by immunoblotting and confirm protein knockdown in the in vitro studies. Whole-cell lysates were prepared by washing the cells three times in PBS, and cells were lysed using radioimmunoprecipitation assay buffer on ice for 30 minutes. These were centrifuged at 13 000 × *g* for 12 minutes at 4°C and the supernatant added to Laemmli buffer. To obtain lysates in nonreducing conditions, the supernatant was added to a nonreducing buffer [250 mM Tris HCl (pH 6.8), with 8% sodium dodecyl sulfate, 40% glycerol, and 0.02% bromophenol blue].

Samples were heated at 95–100°C for 5 minutes and loaded on 4%–12% SDS-PAGE gels. Proteins were then transferred to nitrocellulose membrane (GE Healthcare Life Sciences) using a semidry transfer blot (Bio-Rad Laboratories) at 15 V for 1 hour. Blots were immunolabeled overnight with a rabbit polyclonal anti-TXNRD2 antibody (Sigma-Aldrich; immunogen incorporating amino acid sequence 203–353 of TXNRD2) at 1:500 dilution, mouse monoclonal glyceraldehyde-3-phosphate dehydrogenase (GAPDH) at 1:5000 dilution (Abcam) as a loading control, or rabbit polyclonal anti-TXNRD1 antibody (Abcam). Rabbit polyclonal anti-Peroxiredoxin III antibody (Proteintech) was used at a 1:400 dilution. Visualization of the proteins was performed using Alexa-fluor 680 and 800 secondary antibodies (Invitrogen) at a 1:5000 dilution and the Li-CoR Odyssey system.

### Quantitative real-time PCR

Total RNA was extracted from cultured H295R cells 10 days after puromycin selection using the RNeasy kit (QIAGEN) according to the manufacturer's protocol. RNA samples were quantified with a spectrophotometer and 1.0 μg of RNA from each sample was reverse transcribed after deoxyribonuclease treatment. Quantitative RT-PCR was set up in triplicate (per sample) on a Stratagene Mx3000P thermocycler using KAPA SYBR Fast quantitative PCR master mix with 200 nM forward and reverse primers targeted to *TXNRD2* or *GAPDH* (primer sequences listed in Supplemental Table 1); giving a total volume of 10 μL. After an initial denaturation step of 3 minutes at 95°C, PCR cycling was performed for 40 cycles of 95°C for 3 seconds, 55°C for 20 seconds, and 72°C for 1 second, followed by one cycle of 1 minute at 95°C, 55°C for 30 seconds, and 95°C for 30 seconds.

The expression of *TXNRD2* and *GAPDH* mRNA was investigated using a panel of cDNAs derived from 11 adult tissues (adrenal cortex, heart, liver, testes, thyroid, lung, kidney, spleen, ovary, brain, and skeletal muscle). Quantitative real-time PCR was performed for each tissue as described above.

### MTS (3-(4,5-Dimethylthiazol-2-yl)-5-(3-carboxymethoxyphenyl)-2-(4-sulfophenyl)-2H-tetrazolium), inner salt assay

The CellTiter96 Aqueous nonradioactive cell proliferation assay (Promega) was used to assess cell viability. Cells were plated at a density of 10 000 cells per well in a 96-well plate on day 0. Forty-eight hours later, standards were established by plating H295R cells at densities ranging between 1000 and 35 000 cells per well, in triplicate. Cells were then incubated for 2 hours in a humidified incubator at 37°C. The CellTiter96 kit was used according to the manufacturer's protocol, and the absorbance of the wells at 490 nm was read at 2.5 hours using an ELISA plate reader. There was no significant difference in absorbance readings, between control and *TXNRD2*-knockdown cells, 0.43A ± 0.03 vs 0.42A ± 0.03 (mean ± SD, n = 6).

### Reduced glutathione (GSH)/oxidized glutathione (GSSG) measurements

Measurement of total glutathione (GSH + GSSG) or GSSG was performed using GSH/GSSG-Glo Assay (Promega), a luminescence-based system according to the manufacturer's instructions. Briefly, control and *TXNRD2*-KD cells were plated on a 96-well plate at a density of 20 000 cells/well, and duplicate samples were assayed for total glutathione or GSSG. GSH to GSSG ratios were calculated directly from Net RLU (relative light units) measurements using the equation GSH to GSSG ratio = [net total glutathione RLU-Net GSSG RLU]/[Net GSSG RLU/2].

### Flow cytometry with MitoSOX

Cells were grown to 50%–70% confluency and fresh media added before each experiment. MitoSOX Red (Invitrogen) was added to a final concentration of 5 μM according to the manufacturer's recommendation. After 20 minutes of loading of MitoSOX, *TXNRD2*-knockdown and control cells were trypsinized for 4 minutes and neutralized with media. The cells were washed with PBS (with Ca/Mg) and resuspended in fresh media in a sterile fluorescence-activated cell sorting tube.

For the determination of mitochondrial superoxide by flow cytometry, measurements were carried out using an LSR Fortessa (BD Bioscience), data obtained were recorded and subsequently analyzed using DIVA version 6.2 (BD Bioscience,). Ten thousand gated events were recorded. The following steps were carried out for gating: cell debris as represented by distinct low forward and side scatter were gated out for analysis (P1), only singlet events were gated (P2), GFP-positive cells (cells incorporating either scrambled control or *TXNRD2* knockdown shRNA) were selected (excited by 488 nm blue laser, band pass filter 530/30 nm) (P3), and finally MitoSOX Red was excited by 561 nm yellow/green laser with a band pass filter of 670/30 nm (P4) (Supplemental Figure 1). For quantitative analysis the frequency of events in P4 was multiplied by the median fluorescence intensity (MFI) within P4 to give an integrated MFI (iMFI) (6), reflecting the total functional response of this population of cells to MitoSOX). A Student's *t* test was used for statistical analysis.

## Results

## Case reports

Affected individuals within the kindred exhibited a wide spectrum of severity in symptoms at diagnosis with late onset in several family members (Table 1). The index case (patient 1.1), whose parents were first cousins, was diagnosed with isolated glucocorticoid deficiency at the age of 10.8 years after hyperpigmentation during febrile illnesses. Biochemical results revealed ACTH resistance: raised 9:00 am plasma ACTH (160 ng/L, normal < 50) and low 9:00 am serum cortisol levels (<10 nmol/L, normal range 200–600). Her sister (patient 1.2) was subsequently diagnosed, aged 4.5 years, with a 2-year preceding history of hyperpigmentation.

**Table 1.** Clinical Details of the Members of the Kindred

Patient	Sex	Age, y	Age at Diagnosis, y	Mode of Presentation	Relevant Clinical History	Degree of Pigmentation at Presentation	9:00 am Cortisol, nmol/L	9:00 am ACTH, ng/L	Maximum Cortisol With ACTH Stimulation, nmol/L	Echo
1.1	F	33.8	10.8	Hyperpigmentation	Asymptomatic until diagnosis	Moderate	<10	160	Not done	Normal
1.2	F	27.1	4.5	Hyperpigmentation	Asymptomatic until diagnosis	Severe	<25	500	<25	Trivial TR and MR
2.1	M	13.9	2.9	Screening	Mild neonatal jaundice	None	65	8130	61	Normal
2.2	F	9.5	6.9	Screening	Asymptomatic	Mild	158	514	33	Normal
2.3	F	8.6	0.1	Screening	Asymptomatic	Severe	28	3249	147	Normal
2.4	F	7.4		Currently well	Asymptomatic	None	262^a^	23.2^a^	1052^a^	Normal
2.5	M	2.1	0.1	Poor feeding	Heart failure secondary to cardiac defect	Mild	46	>1240	190	Truncus arteriosus and VSD

Abbreviations: Echo, echocardiogram; F, female; M, male; MR, mitral regurgitation; TR, tricuspid regurgitation; VSD, ventricular septal defect. The 9:00 am cortisol normal range is 200–600 nmol/L, 9:00 am ACTH normal range is less than 50 ng/L. The maximum cortisol with ACTH stimulation normal is greater than 550 nmol/L. Biochemical data are at time of diagnosis.

a Most recent levels.

The children of the index case (patients 2.1–2.3) were screened from birth and diagnosed with glucocorticoid deficiency between the ages of 0.1 and 6.9 years. Patient 2.5 presented at 0.1 year with cardiac failure secondary to congenital truncus arteriosus and a ventricular septal defect. During his admission he was diagnosed with isolated glucocorticoid deficiency. He is the only affected member of the kindred known to have comorbidity. Echocardiograms and electrocardiograms were normal in all individuals homozygous for the mutation except patient 1.2, who has trivial tricuspid and mitral valve regurgitation. All clinically affected individuals demonstrated a poor cortisol response to ACTH stimulation [125 μg tetracosactide (Synacthen) im] requiring standard glucocorticoid replacement therapy. All have normal mineralocorticoid production. Patient 2.4, homozygous for the *TXNRD2* mutation, is currently clinically well aged 7.4 years with normal biochemistry and is under close clinical surveillance. Interestingly, she has had raised ACTH levels in early infancy (9:00 am ACTH of 124 ng/L at 0.02 y of age; corresponding cortisol of 305 nmol/L), which subsequently normalized. Her older sister, individual 2.2, similarly had raised ACTH levels in infancy (9:00 am ACTH 171 ng/L at 0.98 y of age; corresponding cortisol of 448 nmol/L), which normalized, although she was later diagnosed with FGD at 6.9 years.

Affected individuals were mutation negative for the known genetic causes of FGD. Whole-exome sequencing of three affected individuals (denoted by the asterisks in the pedigree, Figure 1A) identified four rare homozygous variants within coding sequences, common to all three individuals, after application of our filtration strategy (see *Materials and Methods*). Three variants in two candidate genes did not segregate with the disease. All three variants are in the Single Nucleotide Polymorphism Database (dbSNP); the *OR2T35* variant (rs370874670) and the two *MUC4* variants (rs374495657 and rs202060675), but there is no frequency or population data. The variants were discounted on the basis that disease affected patient 2.2 and was wild type for rs370874670 in *OR2T35*, and the unaffected husband of patient 1.1 (individual 1.3) was homozygous for both changes in *MUC4*. Only one variant, a stop gain mutation (c.1341T>G; p.Y447X) within exon 15 of *TXNRD2* (RefSeq accession number NM_006440.3), encoding mitochondrial TXNRD2, segregated with the disease in this kindred (Figure 1, A–C). Individuals heterozygous for the change were clinically unaffected.

Two SNPs at this position are annotated in dbSNP (identification rs202059967): an A>G change and the A>C change reported here. The A>G change is silent and seen in only 1 of 12 686 alleles in National Heart, Lung, and Blood Institute Gene Ontology Exome Sequencing Project, ESP6500 (see web site addresses); the A>C change is not recorded in this database but is listed in dbSNP submitted by EXOME_CHIP (submitter SNP identification ss491568437), with no frequency data. Sequencing of more than 1000 healthy adult British Pakistanis revealed a minor allele frequency of 1.04% for this variant, and the genotypes were A/A = 1080; A/C = 23; C/C = 0. Importantly, in this control population, the variant was never seen in homozygosity. One other stop gain mutation (R441*; rs200162480) is listed for *TXNRD2* but is present in heterozygosity in only one individual (1 of 12 730 alleles). Sequencing of 50 FGD patients with unknown etiology identified variants at rs5748469 (p.A66S), rs5992495 (p.S299R), and rs1139793 (p.I370T) with minor allele frequency of 30%, 17.5%, and 25%, respectively, in keeping with their frequencies in the European American cohort from *GO-ESP*. This would fit with the fact that most of our FGD patients are of European extraction. No other variants were discovered in 100 FGD alleles, making the *TXNRD2* mutation a rare cause of FGD.

TXNRD2 is a dimeric NADPH-dependent flavin adenine dinucleotide-containing enzyme that catalyzes the reduction of the active disulphide of thioredoxin 2 and other substrates (7). As a selenoprotein, it requires insertion of a selenocysteine residue for catalytic activity. The mutation was predicted to cause a protein truncation prior to the selenocysteine active site. However, Western blotting of patient lysates revealed absence of the protein in homozygous patients in comparison with a heterozygote carrier and control (Figure 1D). In contrast, patients express cytoplasmic TXNRD1 normally (Figure 1D). Absence of *TXNRD2* cDNA on RT-PCR was consistent with nonsense-mediated decay (Figure 1E), with direct sequencing of the amplicon from a heterozygote carrier revealing amplification of the wild-type sequence alone.

**Figure 2.**
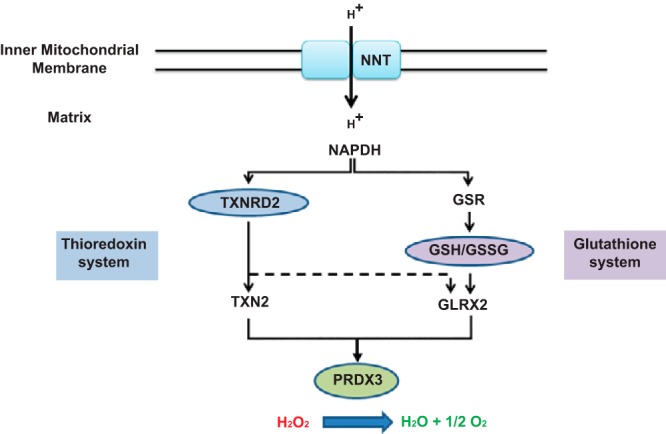
The thioredoxin and glutathione systems maintain mitochondrial redox homeostasis. TXNRD2 reduces TXN2 and GLRX2, both of which can reduce PRDX3, which in turn detoxifies H_2_O_2_ in mitochondria. Glutathione reductase (GSR) and GSH contribute to the process through the reduction of GLRX2. NNT provides the high concentrations of NADPH required by both the thioredoxin and glutathione systems.

**Figure 3.**
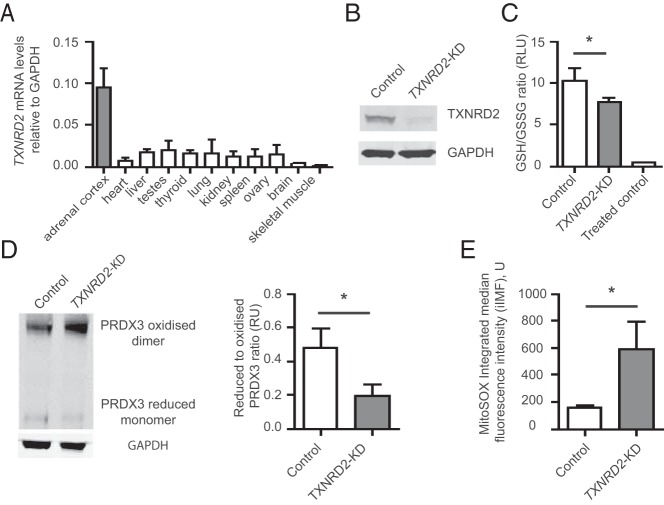
Knockdown of *TXNRD2* impairs mitochondrial redox homeostasis in a human adrenocortical cell line. A, TXNRD2 is widely expressed in human tissues with highest the *TXNRD2* mRNA levels in the adrenal cortex. B, Stable *TXNRD2* knockdown (*TXNRD2*-KD) in H295R cells, confirmed by Western blot analysis with 95% knockdown of protein levels (n = 4). C, Increased pressure on the glutathione system is observed with a significant decrease in the reduced:oxidized glutathione (GSH:GSSG) ratio. Oxidative stress induced by 25 μM menadione in the control cells reduced this ratio to 0.4 ± 0.1 (n = 4). D, *TXNRD2*-KD leads to a decrease in the reduced to oxidized PRDX3 ratio [Western blot with densitometric analysis (n = 3)]. E, Quantitative analysis of superoxide production by MitoSOX, after fluorescence-activated cell sorting (FACS) shows a significant increase in superoxide production in KD cells relative to controls (n = 3). Error bars represent SD. *, *P* < .05.

The glutathione and thioredoxin systems maintain reduced peroxiredoxin 3 (PRDX3) (Figure 2), which is integral for redox regulation within the adrenal (8). NNT provides the thioredoxin and glutathione systems with high concentrations of NADPH required for this process. We found that although *TXNRD2* mRNA is ubiquitously expressed in the human tissues tested, the highest levels are observed in the adrenal cortex (Figure 3A), consistent with mouse expression profiles (9).

*TXNRD2* knockdown in the H295R human adrenocortical cell line by shRNA (Figure 3B) had no effect on cell viability. However, a clear impact on mitochondrial redox homeostasis was demonstrated, with increased pressure on the glutathione system observed as a decrease in the GSH to GSSG ratio (10.3 ± 1.3 vs 7.7 ± 0.4 in scrambled vs knockdown cells, respectively; *P* = .02) (Figure 3C). The ability to maintain mitochondrial PRDX3 in its reduced form is impaired, with a significant decrease in the ratio of the monomeric reduced to oxidized dimeric form in the TXNRD2-deficient cells compared with controls (0.19 ± 0.06 vs 0.48 ± 0.09, respectively; *P* = .02; Figure 3D). Finally, as a consequence of *TXNRD2* knockdown in the adrenocortical cells, an approximately 3-fold increase in levels of mitochondrial reactive oxygen species are seen, further demonstrating an impairment of redox regulation (Figure 3E and Supplemental Figure 1).

## Discussion

Absence of TXNRD2 is associated with adrenal insufficiency in this consanguineous kindred, with all affected members being homozygous for the identified genetic defect. TXNRD2 is 1 of 25 human selenoproteins, which require insertion of a highly reactive selenocysteine residue for enzymatic activity (10). Several selenoproteins, including the thioredoxin reductases and glutathione peroxidases, contribute significantly to redox regulation. TXNRD2, one of three thioredoxin reductases, is mitochondria specific and exists as an antiparallel homodimer (7). The N- and C-terminal redox active centers of the two subunits functionally interact and transfer electrons from NADPH/H^+^ to thioredoxin 2 (TXN2) and other substrates (7). Within the mitochondria the thioredoxin and glutathione systems, reliant on the provision of NADPH/H^+^ by NNT, contribute to the maintenance of redox homeostasis.

Particularly high *TXNRD2* mRNA levels were noted in the adrenal cortex compared with the other human tissues investigated, suggesting a critical role in this tissue. PRDX3 is reported to be the most important H_2_O_2_-eliminating enzyme in the mitochondria of the adrenal cortex with hyperoxidation of PRDX3 resulting in diminished steroidogenesis (8). During H_2_O_2_ elimination, two reduced PRDX3 subunits are converted to an oxidized disulphide-linked dimer that is reduced again by TXN2 (8). Thus, PRDX3, together with mitochondrial-specific TXN2 and TXNRD2, provide a primary line of defense against H_2_O_2_ produced by the mitochondrial respiratory chain in the adrenal gland. Additionally, glutaredoxin 2 has recently been identified as another electron donor for PRDX3 (11). Glutaredoxin 2 (GLRX2) itself is reduced by TXNRD2 as well as GSH, and the mitochondrial thioredoxin and GSH systems function in parallel to protect against oxidative stress (11, 12) (Figure 2). In our in vitro knockdown adrenocortical model, we demonstrate that the glutathione system is unable to fully compensate for the TXNRD2 deficiency leading to increased mitochondrial superoxide production.

Oxidative stress impedes steroidogenesis, and steroidogenesis itself induces oxidative stress as a result of electron leak throughout the steroidogenic pathway (13, 14). The final step of cortisol production, catalyzed by CYP11B1 within the mitochondria, accounts for approximately 40% of the total electron flow from NAPDH directed at reactive oxygen species production during steroidogenesis (14). This, together with the higher production of cortisol in comparison with aldosterone, may explain the particular susceptibility of the zona fasciculata to oxidative stress, and hence, individuals with *TXNRD2* and *NNT* mutations primarily develop glucocorticoid deficiency. Oxidative stress has been implicated in other causes of adrenal insufficiency, including triple A syndrome, in which failure of nuclear import of DNA repair proteins and ferritin heavy chain are described (15, 16) and adrenoleukodystrophy, which is secondary to the accumulation of very-long chain fatty acids in the adrenal cortex and other tissues (17). The wide variability in age at diagnosis (0.1–10.8 y) within this family may suggest the existence of disease modifiers, which could be genetic, environmental, or both. Individual 2.4 currently shows no sign of adrenal disease, which may be due to incomplete penetrance or variable expressivity of the mutation. Because she is only 7.4 years old and because she has manifested abnormal ACTH readings in the past, similar to the clinical picture for her older sister (individual 2.2), she may go on to develop the disease.

Because *TXNRD2* is ubiquitously expressed in humans, individuals with this genetic defect are potentially at risk of developing extraadrenal manifestations. *Txnrd2* deletion in mice is embryonically lethal at day 13 as a consequence of a combination of cardiac and hematopoietic defects, with cardiac-specific ablation resulting in a fatal dilated cardiomyopathy (18). Thus, in mice the TXNRD2 system is clearly indispensable for normal cardiac development and function. In humans, two novel heterozygous mutations in *TXNRD2* were identified in 3 of 227 patients with a diagnosis of dilated cardiomyopathy (19). Interestingly, neither the heterozygote nor homozygote individual, with absence of TXNRD2, showed any evidence of cardiomyopathy or conduction disease. One affected family member presented with truncus arteriosus, an extremely rare congenital cardiac anomaly. Approximately 40% of truncus arteriosus cases are associated with Di George syndrome (DGS), secondary to haploinsufficiency of a region of varying length on chromosome 22q11.2 (20). Several genes within this region have been linked to defects in cardiac development including *TBX1* and *Crkl*, essential for the survival, proliferation, and migration of neural crest cells (21, 22). Because *TXNRD2* falls within this region on chromosome 22, this raises the possibility that TXNRD2 contributes to the cardiac phenotype of DGS. Because heterozygote carriers of the p.Y447X *TXNRD2* mutation have normal adrenal function, we would predict that haploinsufficiency of *TXNRD2* would not lead to an abnormal adrenal phenotype, and consistent with this, we were unable to identify any published reports of adrenal insufficiency in DGS.

We report the first mutation in *TXNRD2* associated with an adrenal phenotype in humans, signifying the importance of the thioredoxin system in maintaining redox homeostasis in the adrenocortical environment. The *TXNRD2* mutation in this family and the other recently described FGD syndromes due to mutations in *NNT* and *MCM4* highlight important pathogenic pathways in addition to defective ACTH signaling, causing glucocorticoid deficiency. A delicate balance of mitochondrial redox regulation controls steroidogenesis in the human adrenal gland (8, 23, 24), and therefore, other components of this complex network of mitochondrial antioxidants make good candidates for undiagnosed causes of adrenal insufficiency.
